# Splenic SUMO1 controls systemic inflammation in experimental sepsis

**DOI:** 10.3389/fimmu.2023.1200939

**Published:** 2023-07-13

**Authors:** Ayman Youssef, Bilal Khan Mohammed, Abhishek Prasad, Angela del Aguila, Gabriel Bassi, Wei Yang, Luis Ulloa

**Affiliations:** Center for Perioperative Organ Protection, Department of Anesthesiology, Duke University Medical Center, Durham, NC, United States

**Keywords:** sepsis, SUMO, TAK981, spleen, infection, organ function

## Abstract

**Introduction:**

The recent discovery of TAK981(Subasumstat), the first-in-class selective inhibitor of SUMOylation, enables new immune treatments. TAK981 is already in clinical trials to potentiate immunotherapy in metastatic tumors and hematologic malignancies. Cancer patients have more than ten times higher risk of infections, but the effects of TAK981 in sepsis are unknown and previous studies on SUMO in infections are conflicting.

**Methods:**

We used TAK981 in two sepsis models; polymicrobial peritonitis (CLP) and LPS endotoxemia. Splenectomy was done in both models to study the role of spleen. Western blotting of SUMO-conjugated proteins in spleen lysates was done. Global SUMO1 and SUMO3 knockout mice were used to study the specific SUMO regulation of inflammation in LPS endotoxemia. Splenocytes adoptive transfer was done from SUMO knockouts to wild type mice to study the role of spleen SUMOylation in experimental sepsis.

**Results and discussion:**

Here, we report that inhibition of SUMOylation with TAK981 improved survival in mild polymicrobial peritonitis by enhancing innate immune responses and peritoneal bacterial clearance. Thus, we focused on the effects of TAK981 on the immune responses to bacterial endotoxin, showing that TAK981 enhanced early TNFα production but did not affect the resolution of inflammation. Splenectomy decreased serum TNFα levels by nearly 60% and TAK981-induced TNFα responses. In the spleen, endotoxemia induced a distinct temporal and substrate specificity for SUMO1 and SUMO2/3, and both were inhibited by TAK981. Global genetic depletion of SUMO1, but not SUMO3, enhanced TNFα production and metabolic acidosis. The transfer of SUMO1-null, but not wild-type, splenocytes into splenectomized wild-type mice exacerbated TNFα production and metabolic acidosis in endotoxemia.

**Conclusion:**

These results suggest that specific regulation of splenic SUMO1 can modulate immune and metabolic responses to bacterial infection.

## Introduction

The innate immune system orchestrates inflammatory responses to fight multiple disorders such as infections and cancer, but unregulated inflammation can become more dangerous than the original onset compromising vital organs (1–3). A typical example is severe sepsis, the most common cause of death in hospitalized patients killing over 250,000 Americans each year (4, 5). Sepsis is characterized by systemic inflammatory response syndrome (SIRS) and multi-organ dysfunction syndrome (MODS) (6). In both syndromes, early TNFα production spreads in the bloodstream triggering systemic inflammation and septic shock (7–9). TNFα blockade prevents the most significant events associated with sepsis, including systemic inflammation, septic shock (7–10), metabolic acidosis (11), and kidney injury (11–13). Metabolic acidoses are categorized into high- or non-anion gap metabolic acidosis, to determine the etiology and hence appropriate treatment. High anion gap metabolic acidosis (HAGMA) is the dominant blood gas anomaly in sepsis (14), and it is mostly due to ketoacidosis, lactic acidosis, or acute kidney injury (AKI). AKI is a life-threatening complication associated with higher mortality in sepsis (7), particularly in critically ill (15) and elderly patients (8). Sepsis and septic shock account for over 50% of AKI in ICUs (16). In addition to creatinine, which is a pivotal indicator for AKI prognosis (17), a study with 1505 patients reported that AKI negatively interacts with HAGMA, but not with lactate, as cofounder to worse 28-day mortality (17). Despite these preclinical results, TNFα inhibition failed to improve survival in sepsis clinical trials, in part, because early TNFα production triggers multifactorial onslaughts leading to HAGMA and AKI (18, 19). Thus, there is an urgent unmet need to identify the integral mechanism modulating the production of inflammatory factors in infectious disorders.

Sentrin/Small Ubiquitin-like Modifiers (SUMO) proteins represent an integral system that reprograms cellular proteostasis, signal transduction, transcription, and metabolism during physiologic stress (20–25). SUMOylation refers to the conjugation of the terminal glycine of SUMO to lysine residues, via the formation of an isopeptide bond, in target proteins as a covalent but reversible post-translational modifier to regulate protein function. SUMOylation is increasingly recognized as a key regulator in diseases as it is very responsive to endogenous and environmental stressors (26, 27). SUMOylation is required for embryonic survival, and general inhibition of SUMOylation by genetic ablation of Ubc9 (ubiquitin-conjugating enzyme 9 that functions as the E2-conjugating enzyme required for SUMOylation) causes early embryonic death (28). Thus, the study of SUMOylation has been limited by the lack of specific inhibitors. The recent discovery of the first-in-class selective SUMO inhibitor, TAK981 (that prevents the transfer of SUMO to the E2 conjugating enzyme Ubc9) is providing new clinical opportunities (29–32). TAK981 (Subasumstat) is currently in a dose-escalation Phase 1/2 clinical study to evaluate its safety, tolerability, and pharmacokinetics to potentiate immunotherapies in patients with advanced or metastatic solid tumors or relapsed/refractory hematologic malignancies such as diffuse large B cell lymphoma after CAR T-cells therapy (Clinicaltrials.gov, NCT03648372, Estimated completion date: October 2024) (33). Regulation of the immune system by SUMOylation is not only critical for cancer but also for infections and sepsis. Cancer and chemotherapy patients have more than ten times higher risk for sepsis and around ten times higher mortality than non-cancer patients (34–37). In the current era of widespread antibacterial resistance affecting septic patients, there is an urgent need for new treatments to modulate the immune system (38–40). However, the effects of TAK981 on infections are unknown, and the previous results on SUMOylation and infections are conflicting (20). Genetic depletion of Ubc9 in myeloid cells renders mice more resistant to viral infections, but inhibition of Ubc9 in the bone marrow renders mice more susceptible to endotoxemia (24). However, endotoxemia is a simple model to study the immune system without an active pathogen, and the analysis of SUMOylation in infectious disorders was limited by the lack of specific inhibitors. Thus, here we study the role of SUMOylation and TAK981 in both polymicrobial peritonitis and lethal endotoxemia to analyze innate immune responses, bacterial clearance, metabolic acidosis, and organ dysfunction. We also analyzed the specific SUMO isoforms involved in the integral responses to bacterial endotoxin.

## Materials and methods

### Chemicals and reagents

LPS (Escherichia coli 0111:B4) was purchased from Sigma-Aldrich (Saint Louis, MO) and dissolved in sterile pyrogen-free PBS (Gibco, Life Technologies, Grand Island, NY). SUMO inhibitor TAK981 (Subasumstat) is well-described in the literature (29–32), was purchased from ChemieTek (Indianapolis, IN).

### Animal experiments

Animal procedures were approved by the Institutional Animal Care & Use Committee of Duke University. All animal experiments were performed in 6-8-week-old (∼25 ± 5 g) male mice. Wild-type C57BL/6 male mice were obtained from The Jackson Laboratory (Bar Harbor, ME). SUMO1-, SUMO3-knockout and littermates wild-type mice were produced in our labs as we described (41, 42). Knockout mice were genotyped by PCR using tail genomic DNA and the Extract-N-Amp Tissue PCR kit (Sigma Chemical, Saint Louis, MO) as described (41, 42). Mice were maintained on 12h light-dark cycle, with free access to food and water (ad libitum). Mice were randomized into experimental groups and outcome assessors were blinded to group treatments.

### Experimental sepsis

Cecal ligation and puncture (CLP) were performed as we described (43). Mice were anesthetized with 5% isoflurane, shaved, and the lower abdomen was disinfected. A one cm vertical incision was made to expose the cecum. Cecal ligation was done mid-way between the cecal tip and the ileocecal valve using a 6/0 proline. The cecal puncture was done once between the ligature site and cecal tip using a 25-gauge needle and the stool was extruded one mm. Then, the cecum was returned to the abdomen, and the abdominal wall was sutured in layers using a 6/0 proline. Mice were pretreated with vehicle or TAK981 (7.5 mg/Kg;s.c.) 12h before the CLP and every 24h for 4d starting right after the surgery (44, 45). Survival was monitored every 12 hours for two weeks. Endotoxemia was performed using a lethal dose of LPS (7 mg/Kg; i.p.) as we described (43). LPS (*E. coli* LPS 0111:B4; Sigma Chemical, Saint Louis, MO) was dissolved in sterile pyrogen-free PBS (Gibco: Life Technologies, Grand Island, NY) and sonicated for 10 min immediately before use.

### Splenectomy

Splenectomy was performed as we described (46). Mice were anesthetized with isoflurane 5% and subjected to an abdominal incision on the epigastrium and mesogastrium. The spleen was exposed by gentle retraction of the stomach to the side. The three main branches of the splenic artery were stabilized with nylon thread, ligated, and cut. The spleen was removed, and the abdominal wall was sutured with catgut and the skin with nylon thread. Mice were splenectomized 5 days before the experimental procedure and checked daily.

### Cytokine and organ function analyses

Mice were anesthetized by 5% isoflurane and blood was collected by cardiac puncture at the indicated time points, allowed to clot for 1h at room temperature, and centrifuged at 2,000*g* for 20 min at 4°C. Organs were collected at the indicated time points, snap frozen in liquid nitrogen, and homogenized on ice in RIPA buffer supplemented with protease inhibitor (Pierce protease inhibitor mini-tablets, A32955, Thermo Scientific, Rockford, IL) as we described (42). TNFα and IFNγ were analyzed by ELISA (Invitrogen, Life Technologies, NY) as per manufacturer instructions. Blood chemistry was analyzed at 48 hours in blood collected from the vena cava into heparin microtiter tubes and analyzed with CHEM8+ cartridges with the iSTAT1 blood analyzer (Abbott Laboratories, Chicago, IL).

### Peritoneal lavage and bacterial load analysis

Peritoneal lavage was done as previously described (47). Briefly, 24 hours after CLP, mice were anesthetized with 5% isoflurane, the abdominal wall was disinfected with 70% ethanol, and 5 mL of sterile pyrogen-free PBS was injected i.p. using a 25g needle. Then, the abdomen was massaged for 1 minute, and 1 mL of peritoneal fluid was aspirated using a sterile syringe. Peritoneal aspirates were centrifuged at 2,000*g* for 20 min at 4°C and supernatants were frozen at -20 for further cytokines analysis. Peritoneal lavage bacterial load was analyzed as previously described (48). Freshly collected peritoneal aspirates were cultured in blood agar using a sterile inoculation loop and agar plates (Hardy Diagnostics, Santa Maria, CA) and were incubated at 37°C for 24 hours. Bacterial colonies (CFU/mL of peritoneal aspirate) were counted using an ImageJ Fiji software cell analyzer (49).

### Western-blots

Western blot assays were performed as we described (50). The spleens were homogenized (1:10 w/v) in lysis buffer (50mM β-glycerophosphoric acid, disodium salt, 5-hydrate, 1mM EGTA pH 8.0, 0.5 mM Na3VO4 and triton x-100) supplemented with protease inhibitor (Pierce protease inhibitor mini-tablets, A32955, Thermo Scientific, Rockford, IL), 20mM SUMO degradation inhibitor N-ethylmaleimide (Sigma, Saint Louis, MO), and 0.5mM DL-Dithiothreitol (VWR, Radnor, PA), then centrifuged at 16,000*g* for 15min at 4°C. Supernatant protein concentration was determined with Bio-Rad Protein Assay (Bio-Rad, Life Science Research, Hercules, CA). Samples were run on the Bolt Bis-Tris Mini Gels (Novex, Invitrogen, Life Technologies, Grand Island, NY) at 150 V for 50 min. The samples were transferred onto the PVDF membranes (Hybond-P, Amersham, GE Healthcare, Pittsburg, PA) with the Bio-Rad Gel Transfer (Bio-Rad, Life Science Research, Hercules, CA). The membranes were blocked with 5% skimmed milk-TBST for 1 h at 4°C and incubated with antibodies against SUMO1 (1:1,000, PA5-96065, Thermo Scientific, Rockford, IL) or SUMO2/3 (1:1,000, PA5-19418, Thermo Scientific, Rockford, IL) overnight at 4°C. Then, washed in TBST and incubated with HRP-linked antibody to rabbit IgG (1:5,000, G21234, Thermo Scientific, Rockford, IL) for 1h at room temperature. The membranes were washed and processed with chemiluminescence (Amersham, Buckinghamshire, UK) and analyzed with Amersham gel imager 680 (Amersham, Buckinghamshire, UK).

### Splenocytes transfer

Splenocytes were isolated as we described (51). Briefly, harvested spleens were mechanically disrupted using 70-μm nylon mesh, then filtered using a 70μm cell strainer in ice-cold PBS. The suspension was centrifuged 800g for 6 minutes at 4°C, and pellets were resuspended in RBC lysis buffer (Biolegend, Sand Diego, CA) for 5 minutes on ice. Cells were washed twice in ice-cold PBS before resuspending them in Trypan Blue 1:1 for cell counting (Countess™, Invitrogen, Life Technologies, Grand Island, NY). Splenocytes (10x10 (6) live cells/20g mouse) were injected i.p. in splenectomized recipient mice 24 hours before the LPS challenge.

### Statistical analyses

Statistical analyses were performed with GraphPad Prism Software (GraphPad Software, San Diego, CA). The sample size was calculated based on our preliminary results using G*Power3.1 and ANOVA (Fixed effects, omnibus, one-way), assuming effect size= 0.25, α=0.05, power (1-beta err prob)=0.8. Data are expressed as mean ± sem and figures represent experiments repeated twice on different days. The Student’s t-test (Mann-Whitney U test) was used to compare two experimental groups. Three or more groups were analyzed with parametric one-way ANOVA with multiple pair-wise comparisons. Normality and homogeneity of variance were confirmed with Kolmogorov-Smirnov analyses. Pair comparisons in nonparametric ANOVA tests were adjusted *post-hoc* with the Tukey test (in equal sample sizes) or Bonferroni’s for multiple hypothesis testing. The time courses and pair-wise comparisons were analyzed with the two-way ANOVA for repeated measures. Survival was analyzed with the Log-rank (Mantel-Cox) test. Statistical significance was established at p≤.05 with a 2-sided alpha according to Cohen (52).

## Results

### Inhibition of SUMOylation improved survival in polymicrobial peritonitis

**Figure 1:**
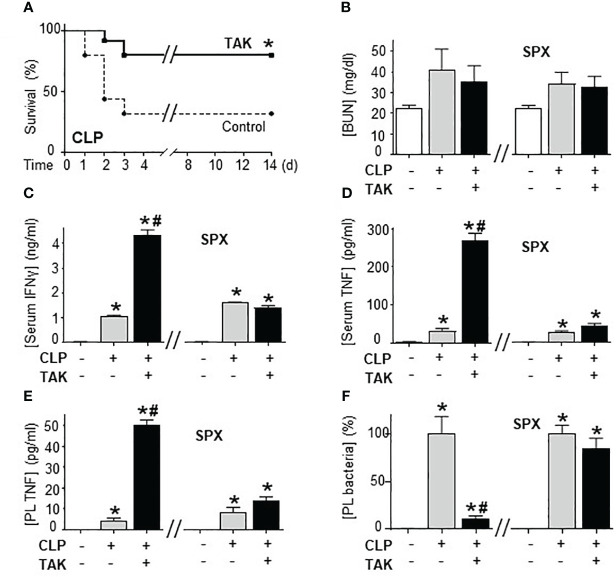
Inhibition of SUMOylation improved survival in polymicrobial peritonitis. **(A)** Kaplan-Meier survival analyses in polymicrobial peritonitis induced by cecal ligation and puncture (CLP) in mice treated with vehicle or TAK981 (7.5 mg/kg; s.c.). *P<0.05 vs. Control, (n=25/group, Survival Log-rank test. Experiment repeated twice at different days). **(B)** blood urea nitrogen (BUN), **(C)** IFNγ, and **(D)** TNFα serum levels in control and septic mice treated with vehicle or TAK981. **(E)** TNFα, and **(F)** bacterial colony forming units in peritoneal lavage (PL) from control and CLP mice with or without splenectomy (spx) treated with vehicle or TAK981. *P<0.05 vs. Control (n=6/group, Two-way ANOVA with Bonferroni’s post-hoc test). ^#^P<0.05 vs. CLP.

**Figure 2:**
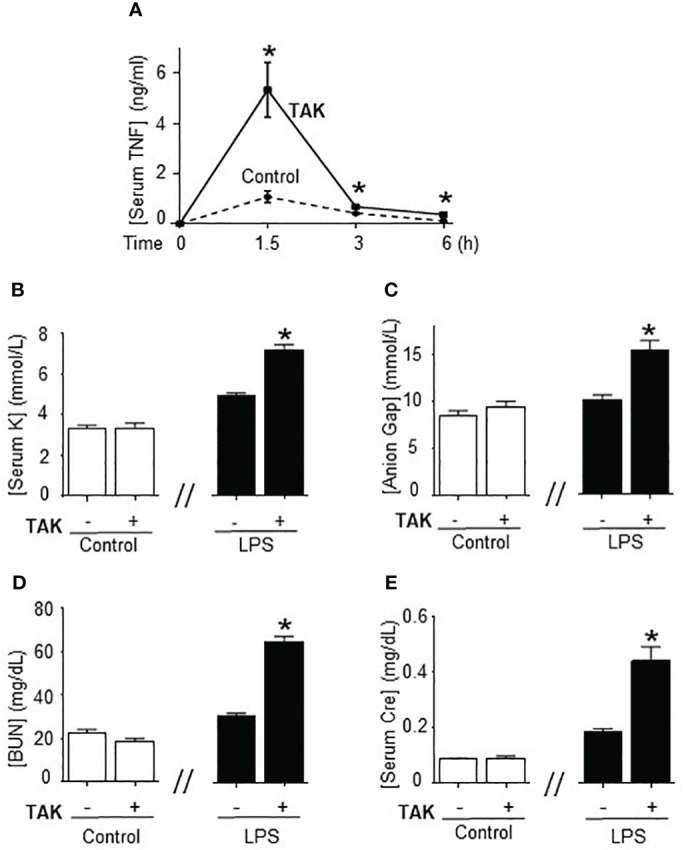
Inhibition of SUMOylation in fatal endotoxemia. **(A)** Time-course of serum TNFa levels in endotoxemic (LPS, 7 mg/kg; i.p.) mice treated with vehicle (control) and TAK981 (7.5 mg/kg; s.c.) *P<0.05 vs. Control (n=6/group, Two-way ANOVA with Bonferroni’s post-hoc test). Serum levels of **(B)** potassium (K), **(C)** anion Gap, **(D)** blood urea nitrogen (BUN), and **(E)** creatinine (Cre) at 48h post-LPS in control and endotoxemic mice treated with vehicle or TAK981. *P<0.05 vs control (n=6/group, unpaired two-tailed t-test).

Previous studies of SUMOylation were performed in endotoxemia, an experimental model that focuses on the immune responses to bacterial endotoxin (LPS) without an active pathogen. Given the clinical implications of sepsis in cancer and that TAK981 is currently in clinical trials for cancer patients, we analyzed the effects of TAK981 on polymicrobial peritonitis. Unlike endotoxemia, cecal ligation and puncture is the most clinically relevant experimental model of sepsis with polymicrobial peritonitis produced by the cecal puncture and necrotic tissue produced by cecal ligation (53–55). TAK981 treatment improved survival in mild polymicrobial peritonitis induced by cecal ligation and puncture (**Figure 1A**). Survival was recorded for over two weeks and no late deaths were observed. In line with previous studies, mild polymicrobial peritonitis induces a mild increase in blood urea nitrogen (**Figure 1B**). Next, we analyzed the effects of TAK981 on the immune responses to bacterial infection. TAK981 treatment did not affect blood urea nitrogen but increased the innate immune responses to bacterial infection including serum IFNγ and TNFα levels (**Figures 1C, D**). TAK981 treatment also increased TNFα levels in peritoneal lavage (**Figure 1E**), and significantly decreased bacteria count in peritoneal lavage of septic mice (**Figure 1F**). Since the spleen is the major source of inflammatory cytokines in experimental sepsis, we repeated the experiments in splenectomized animals to confirm that splenectomy inhibited all the effects induced by TAK981. These results suggest that inhibition of SUMOylation with TAK981 enhances innate immune responses to fight infections. However, these inflammatory responses can become more detrimental than the original infection. Given the complexity of polymicrobial peritonitis, we next focused on the immune and metabolic responses to bacterial endotoxin using experimental models of endotoxemia, which is the most common and reliable model to study the innate immune system (9, 55, 56). TAK981 treatment given at 12h and right before the endotoxemic challenge (LPS, 7 mg/kg; i.p.) did not induce TNFα in control mice but increased TNFα production in lethal endotoxemia (**Figure 2A**). TAK981 increased acute TNFα production but did not affect immune resilience and resolution of serum TNFα levels. Serum TNFα levels peak at 1.5h and quickly resolve by nearly 80% at 3h. Likewise, TAK981 did not affect blood chemistry or anion gap in control mice, but increased hematocrit, hyperkalemia, and high anion gap metabolic acidosis (HAGMA) in lethal endotoxemia (**Supplementary Figure 1A**; **Figures 2B, C**). These effects were specific because TAK981 did not affect natremia, chloremia, or total carbon dioxide (**Supplementary Figures 1B–D**). These results suggested kidney dysfunction, and TAK981 increased blood urea nitrogen and serum creatinine by 25% and 50%, respectively in lethal endotoxemia without affecting control mice (**Figures 2D, E**). These results show that SUMOylation is an integral mechanism that can be exploited to modulate systemic immune responses to bacterial infection. Inhibition of SUMOylation with TAK981 can enhance TNFα responses to fight bacterial infection in mild polymicrobial peritonitis. However, like in clinical sepsis, these immune and metabolic responses can become detrimental in lethal endotoxemia or acute severe sepsis.

### Splenectomy abrogated TAK981 control of systemic inflammation

**Figure 3:**
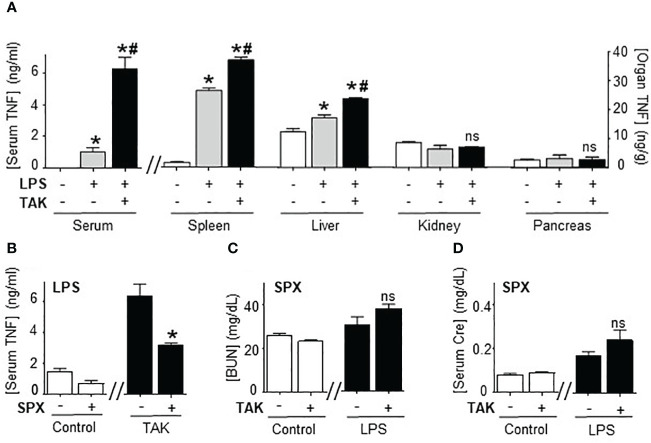
Splenectomy abrogated TAK981 control of systemic inflammation. **(A)** Serum and organ TNFα levels at 1.5h in control and endotoxemic mice treated with vehicle or TAK981. *P<0.05 vs control; ^#^P<0.05 vs LPS (n=6/group, One-way ANOVA with Bonferroni’s post-hoc test). **(B)** Serum TNFα levels in control or TAK981-treated after sham or splenectomy (SPX). **(C)** Blood urea nitrogen (BUN) and **(D)** creatinine (Cre) serum levels at 48h post-LPS in splenectomized (SPX) mice challenged with PBS (control) or LPS and treated with vehicle or TAK981. *P<0.05 vs sham (n=6/group, unpaired two-tailed t-test). ns, Not Significant.

Next, we analyzed how TAK981 modulates systemic immune responses to bacterial endotoxin by analyzing the TNFα levels in different organs. Organ analyses showed the highest TNFα levels in the spleen, and the main effects of TAK981 were increasing TNFα levels specifically in the spleen and liver but not in other organs like the kidney or pancreas (**Figure 3A**). Then, we analyzed the role of the spleen and liver by performing surgical splenectomy. Splenectomy prevented most of TAK981-induced immune and metabolic effects, including TNFα production, hyperkalemia, HAGMA, and kidney dysfunction markers blood urea nitrogen and creatinine (**Figures 3B–D**; **Supplementary Figures 2A–F**). These results show that the immune and metabolic effects induced by TAK981 are mainly mediated by the spleen. Thus, SUMOylation in the spleen appears to reprogram the immune and metabolic responses to bacterial infections.

### Endotoxemia induced a distinct temporal and substrate specificity for splenic SUMO isoforms

**Figure 4:**
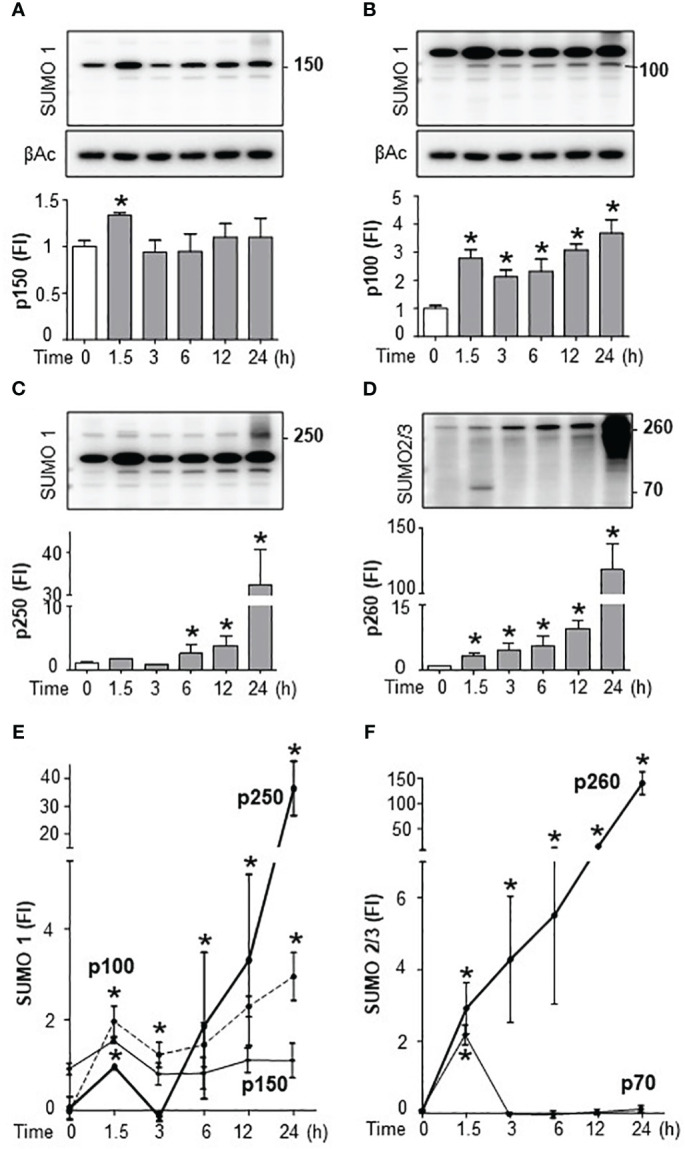
Endotoxemia induced a distinct temporal and substrate specificity for splenic SUMO isoforms. Western-blots (top) and densitometric analyses (bottom) of splenic SUMOylation at the indicated post-LPS time points. Bacterial endotoxin induced SUMOylation-1 of **(A)** p150, **(B)** p100, **(C)** p250, and **(D)** SUMOylation-2/3 of p70 and p260 (smear). Comparative densitometric analyses of **(E)** SUMO1 and **(F)** SUMO2/3 at the indicated post-LPS time-points. β-actin Western-blots (bottom panels) were used as an internal control for protein loading. were used as an internal control for protein loading. Western-blots represent experiments repeated at least twice on different days and graphs show mean ± SEM of fold of induction (FI). *P*<*0.05 vs Control (n=3/group, two-way ANOVA with Tukey’s *post hoc* test).

We next analyzed the specific SUMO isoforms in the spleen by Western blots for SUMO1 and SUMO2/3. SUMO2 and SUMO3 are referred to together as SUMO2/3 because they share ∼95% sequence identity and are undistinguishable by current antibodies (19). Full SUMO1 blots appear to show that the levels of conjugated follow that of non-conjugated SUMO1 suggesting a global increase that could result from increased transcription. However, late conjugated SUMO2/3 levels outweigh non-conjugated levels showing a specific induction of SUMOylation (**Supplementary Figures 3A, B**). Western blots were then analyzed by densitometry for quantification and statistical analyses. Endotoxin increased 2-fold 150KDa SUMO1 signal at 1.5h to return to a normal background at 3h with a slight late secondary increase around 24h (**Figure 4A**; **Supplementary Figure 3A**). Endotoxemia also induced 100KDa SUMO1 signal at 1.5h with a progressive slight increase for up to 24h (**Figure 4B**). These early kinetics of SUMO1 at 100 and 150KDa mimic serum TNFα levels during endotoxemia. However, 150KDa, but not 100KDa, SUMO1 signal was also observed in control mice (without TNFα). Endotoxemia also induced a late 30-fold induction of 250KDa SUMO1 signal at 24 h (**Figure 4C**). SUMO2/3 also has a temporal substrate specificity. Endotoxemia induced an early 70KDa SUMO2/3 signal at 1.5h only (**Figure 4D**; **Supplementary Figure 3B**). 70KDa SUMO2/3 signal was not detected in control mice or late endotoxemia. The most significant effect of endotoxemia was the progressive late smear of 150-260KDa SUMO2/3 signal by 24h (**Figure 4D**). Quantitative comparative analyses show that the most significant effects of endotoxemia were early SUMO1 signals at 100 and 150KDa, and late p250 and early SUMO2/3 signal at 70KDa, and late 260KDa smear (**Figures 4E, F**; **Supplementary Figures 3C, D**). Next, we analyzed the effects of TAK981 on these signals. TAK981 treatment inhibited all SUMOylation as confirmed by Western-blot analyses (**Supplementary Figures 4A, B**). These results show a distinct temporal and substrate specificity for splenic SUMO isoforms in response to bacterial endotoxin.

### Specific inhibition of SUMO1 unfettered TNFα production in endotoxemia

**Figure 5:**
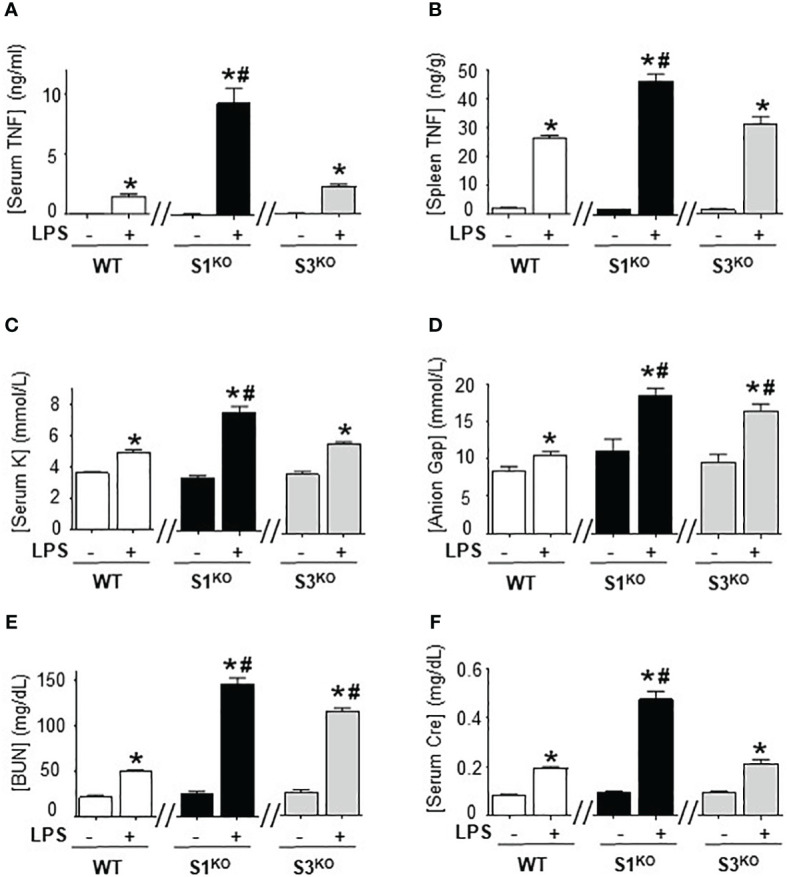
Specific inhibition of SUMO1 unfettered serum TNFα levels in lethal endotoxemia. **(A)** Serum and **(B)** spleen levels of TNFα at 1.5h, and **(C)** sodium (Na), **(D)** anion gap (Gap), **(E)** creatinine (Cre), and **(F)** blood urea nitrogen (BUN) at 48h in wild-type (WT), SUMO1-KO (S1^KO^), and SUMO3-KO (S3^KO^) with vehicle or LPS. *P<0.05 vs control mice without LPS, ^#^P<0.05 vs WT+LPS (n=6/group, unpaired two-tailed t test).

We next determined the specific SUMO isoform regulating immune responses, we bred SUMO1-null and SUMO3-null genetic mutant mice. SUMO1-null and SUMO3-null mice are viable and survive through embryogenesis but SUMO2-null embryos die early during embryonic development and are not viable (41, 42). Wild-type, SUMO1-null, and SUMO3-null mice had nearly undetectable serum TNFα levels in normal conditions, and wild-type and SUMO3-null mice had similar responses to endotoxin (**Figure 5A**). By contrast, SUMO1-null mice exhibit unfettered TNFα production reminiscent of that found with global inhibition of SUMOylation with TAK981. SUMO1-null mice had almost 5-fold higher serum and splenic TNFα levels than wild-type endotoxemic mice (**Figures 5A, B**). These inflammatory responses were specific in the spleen because wild-type, SUMO1-null, and SUMO3-null mice all have statistically similar hepatic TNFα levels in control and endotoxemic mice (**Supplementary Figure 5A**). Thus, specific SUMO1 inhibition unfetters splenic TNFα production and systemic inflammation during lethal endotoxemia.

We also analyzed the specific role of SUMO1 and SUMO2/3 in HAGMA and kidney function markers. Endotoxemia induced similar hyperkalemia and creatinine levels in wild-type and SUMO3-null mice, but the latter had higher HAGMA and blood urea nitrogen (**Figures 5C–F**). Following TNFα responses, SUMO1-null mice also had higher hyperkalemia, HAGMA, blood urea nitrogen, and creatinine levels. Of note, hyperkalemia and creatinine levels mimicked TNFα responses, but not in SUMO3-null mice as they had similar TNFα responses than wild-type mice, but higher HAGMA and blood urea nitrogen. These results are specific to these markers and no differences were observed in hematocrit, natremia, or chloremia between wild-type, SUMO1-null, and SUMO3-null mice (**Supplementary Figures 3B-E**). These results reveal a different role of SUMO1 and SUMO3 in regulating the immune and metabolic responses to lethal endotoxemia and show that specific SUMO1 inhibition increased serum TNFα levels and HAGMA.

### Adoptive transfer of SUMO1-null splenocytes unfettered TNFα production

**Figure 6:**
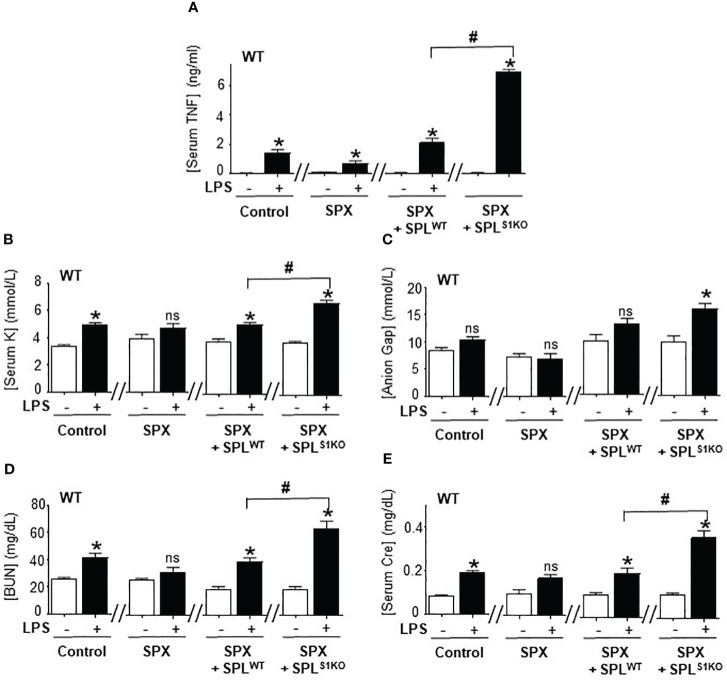
Adoptive transfer of SUMO1-null splenocytes enhanced inflammatory responses. Serum **(A)** TNFα at 1.5h post-LPS and, **(B)** potassium **(K)**, **(C)** anion Gap, **(D)** blood urea nitrogen (BUN), and **(E)** creatinine (Cre) at 48h post-LPS in control and endotoxemic mice after sham (control), splenectomy (SPX), and transfer of splenocytes from wild-type (SPL^WT^) or SUMO1-KO (SPL^S1KO^) mice. *P<0.05 vs control mice without LPS, ^#^P<0.05 vs SPX+ SPL^WT^+LPS (n=6/group, unpaired two-tailed t test). ns, Not Significant.

We further analyzed the role of splenic SUMOylation with the adoptive transfer of splenocytes from SUMO1-null and SUMO3-null mice into splenectomized wild-type mice. Splenectomy attenuated serum TNFα levels and metabolic acidosis in lethal endotoxemia (**Figures 6A–E**). Then, we performed a dose-response curve to determine whether the transfer of wild-type splenocytes into splenectomized mice restores TNFα responses to endotoxin. The adoptive transfer of 1x10 (7) splenocytes intraperitoneally restored typical TNFα responses, HAGMA (hyperkalemia and anion gap), and kidney dysfunction (blood urea nitrogen and creatinine) in splenectomized endotoxemic mice (**Figures 6A–E**). These results were specific because the transferred splenocytes neither induced an effect by themselves nor affected the other markers, including hematocrit, natremia, or chloremia in control or endotoxic splenectomized mice. The transfer of a higher number of splenocytes increased these responses in a concentration-dependent manner and started to induce unspecific responses. Next, we analyzed the role of SUMO1-null splenocytes by transferring splenocytes from SUMO1-null mice into wild-type splenectomized mice. Adoptive transfer of the of 1x10 (7) SUMO1-null splenocytes dramatically increased TNFα responses to bacterial endotoxin in splenectomized wild-type mice by 3-fold higher than the transfer of wild-type splenocytes (**Figure 6A**). This higher TNFα production induced by SUMO1-null splenocytes also triggered the responses observed in SUMO1-null mice, including higher hyperkalemia and anion gap levels. The transfer of SUMO1-null splenocytes also increased both blood urea nitrogen and creatinine in endotoxemic mice (**Figures 6D, E**). These results show that the specific transfer of SUMO1-null splenocytes is sufficient to recapitulate the immune and metabolic responses to lethal endotoxemia observed in SUMO1-KO mice.

## Discussion

The clinical implications of SUMOylation became more relevant considering the link between cancer and sepsis, both resulting from a dysregulated immune system. SUMOylation has been associated with advanced staging and high-risk genetic mutations like c-Myc rearrangement and high-grade B-cell Non-Hodgkin lymphoma (NHL) (26, 57). Conversely, inhibition of SUMOylation provided promising results in pre-clinical cancer models, and depletion of Ubc9 or SUMO-activating enzyme subunit 2 (SAE2) inactivated c-Myc oncogenic mutations and enhanced immune and chemotherapy sensitivity in small cell lung cancer (26–58). The recent discovery of TAK981 enables new immune and antineoplastic treatments. TAK981 is already in clinical trials to potentiate immunotherapies in metastatic solid tumors and refractory hematologic malignancies such as large B cell lymphoma after CAR T-cells therapy. However, the effects of TAK981 in infectious disorders and sepsis are unknown, and the previous results of SUMOylation in infection are conflicting (20). Genetic depletion of Ubc9 in myeloid cells renders mice more resistant to viral infections, but Ubc9 depletion in bone marrow renders mice more susceptible to endotoxemia (24, 55, 59). One critical consideration is that endotoxemia activates the innate immune system without an active pathogen, and does not reflect the role of SUMOylation in bacterial infection. We first studied the effects of TAK981 in polymicrobial peritonitis induced by cecal ligation and puncture, the most clinically relevant experimental model of sepsis with a polymicrobial infection (53–55). TAK981 treatment improved survival in mild polymicrobial peritonitis without antibiotics. These results resemble the protection against viral infections induced by Ubc9-depletion in myeloid cells. Our results also show that TAK981 treatment enhanced immune responses increasing both IFNγ and TNFα serum levels. The increase of IFNγ in our polymicrobial peritonitis is like that described in viral infections induced by genetic depletion of Ubc9 in myeloid cells. We also observed that TAK981 increased TNFα levels and decreased bacterial counts in peritoneal lavage. Together, these results suggest that inhibition of SUMOylation with TAK981 potentiates immune responses that protect mice against mild bacterial infections like those described in viral infections. Whereas Ubc9 depletion increases protective IFNγ responses to viral infections, TAK981 increases TNFα anti-bacterial responses. However, these protective responses can turn detrimental when endotoxemia, bacterial, or viral infections are overwhelming unleashing overzealous responses that became pathological onslaughts. A significant example in our results is that TAK981 increased kidney dysfunction in lethal endotoxemia but not in mild polymicrobial peritonitis because the increased TNFα (∼300 pg/serum mL) responses in CLP mice were not as strong as that observed in severe lethal endotoxemia (∼5,000 pg/serum mL). Overzealous inflammatory responses are equally detrimental in bacterial and viral infections such as COVID-19, and multiple viruses benefit from impaired SUMOylation. For instance, HPV16/18 E6 oncoprotein mediates proteasomal degradation of Ubc9 to promote viral replication and cervical cancer (60). HSV1 ICP0 protein inhibits global SUMOylation to suppress intrinsic immunity for viral replication and tumor suppressor promyelocytic leukemia protein (61). Thus, SUMOylation appears to be an integral mechanism for reprograming protective immune and metabolic responses to infections that can become detrimental. SUMOylation’s clinical implications depend on the infection’s magnitude and type and represent a critical link between immunity, cancer, and infectious disorders.

Previous studies show that Ubc9 depletion increases protective IFN1 responses to viral infections, and unleashes massive nearly 6-fold induction of IL6 responses (∼60ng/ml serum) to bacterial endotoxin at 6h post-LPS (24). Although these IL6 levels are remarkably high as compared to patients with sepsis (<1 ng/ml) or septic shock (up to 10 ng/ml) (62), they suggest a critical role of IL6 in lethal endotoxemia. These studies also report a dramatically lower increase of TNFα (∼125pg/ml serum) that appears to be balanced with stronger IL10 production (∼400pg/ml serum) (24). Our results show a critical role of early acute TNFα production within the first hour after the infection before the reported IL6 and IL10 production in both experimental and clinical studies (9, 63). However, acute TNFα production returns to basal levels around 3h post-LPS showing that inhibition of SUMOylation increased TNFα production but did not affect immune resilience. By contrast, Ubc9 depletion not only increased IFN1 production but held transcription, sustaining its production, and impaired the resolution of IFN1 responses. TNFα was originally defined based on its ability to induce hemorrhagic necrosis of transplanted mouse tumors and by its selective cytotoxicity for transformed cells (64, 65). TNFα-null mice are highly susceptible to infectious agents like *Candida* with a delayed resolution of the *C. parvum*-induced inflammatory responses, but they are resistant to lethal endotoxemia (66, 67). However, cytokine production induced by LPS appears essentially intact in TNFα-null mice, except for reduced colony-stimulating factor activity (66). Our results resemble the clinical time course analyses in septic patients. TNFα appears to play a critical role in sepsis and septic shock: TNFα seems to be sufficient and necessary for “*septic shock*” because (a) TNFα is found in patients and experimental models of “septic shock”, (b) TNFα administration causes cardiovascular shock, hypotension, intravascular coagulopathy observed in “septic shock”; and (c) TNFα neutralization prevents endotoxic- and bacteremia-induced shock (7–10). TNFα blockade prevents endotoxic- and bacteremia-induced shock (7–10) and attenuates metabolic acidosis and pulmonary dysfunction in sepsis (11), the most significant events associated with sepsis (13, 55, 59). Furthermore, TNFα is not only considered an immune cytokine but also a critical metabolic messenger (13). Given the pleiotropic potential of TNFα, it is not surprising that higher TNFα production can enhance later IL6 production, metabolic acidosis, and kidney injury. However, SUMOylation not only regulates TNFα production but multiple immune and metabolic factors. Our results show a strong correlation between early TNFα production, metabolic acidosis, and kidney injury in lethal endotoxemia. Together, these results suggest that inhibition of SUMOylation may provide resistance against viral infections inducing IFN1, and protection against polymicrobial peritonitis by inducing early TNFα production.

One critical limitation of previous studies is analyzing global SUMOylation because mammals express five SUMO isoforms. SUMO1-3 are ubiquitously expressed whereas SUMO4 mRNA was found only in the spleen, kidney, and lymphatic nodes, and SUMO5 is expressed in testes and peripheral leukocytes (68). Our results show that splenectomy prevented most TAK981-induced effects. Although our splenectomy may affect peritoneal immune components, these results concur with our previous studies showing that the spleen is a major source of inflammatory cytokines in systemic inflammation and splenectomy inhibits the production of inflammatory cytokines such as TNFα by over 60% as we previously reported (51, 69–71). These results have also significant clinical implications as the most dreadful complication of splenectomy is overwhelming post-splenectomy infection (OPSI), which is associated with higher morbidity and mortality rates (71, 72). However, future studies will require to determine how splenectomy may affect peritoneal immune components. We next focused on the analyses of splenic SUMOylation. SUMO4 protein has not been detected *in vivo*, and the *sumo4* gene encodes a structurally inactive SUMO (73), and it is considered a pseudogene lacking introns (74, 75). Thus, splenic SUMOylation controlling TNFα production must be controlled by SUMO1-3. Next, we observed that TAK981 inhibits both SUMO1 and SUMO2/3. SUMO2 and SUMO3 are typically referred to as SUMO2/3 because they share ∼95% sequence identity, differ in three amino acids only, are assumed functionally redundant, and are indistinguishable from current antibodies. However, global deletion of *Sumo2* is embryonically lethal, but SUMO1 and SUMO3-null mice are viable without obvious phenotype (41, 42). Some authors consider these results to be due to the lower expression of SUMO3 (as compared to SUMO2) unable to compensate for the massive loss of SUMO2 and not to different biological functions. Full SUMO blots suggest a differential regulation during endotoxemia, with similar levels of conjugated and non-conjugated SUMO1 suggesting a global increase likely due to increased transcription, whereas late conjugated SUMO2/3 levels outweigh non-conjugated levels showing a specific induction of SUMOylation. Specific band and time course analyses of splenic SUMOylation reveal a distinct pattern for SUMO1 and SUMO2/3 without a significant overlapping. Although 250KDa SUMOylation at 24h might represent a potential overlapping between SUMO1 and SUMO2/3, it is not clear whether SUMO2/3 smear is due to different poly-SUMOylation levels of the same or different proteins, and SUMO1 shows a very discrete band suggesting one single substrate. Our results also show that the typical SUMO smear in Western blots depends on the time, cell type, and physiological stage. We observed the typical SUMO2/3 smear at late time points after 12h at 24h. Likewise, our results also show SUMO1 smear at late time points and higher molecular weights. The smear is significantly higher in SUMO2/3, because they are more expressed at higher levels and because they have consensus SUMOylation sites that enable self-SUMOylation to form polymeric chains, but SUMO1 conjugates as a monomer (76–78). Some authors proposed that SUMO1 could serve as a chain terminator of SUMO2/3 polymers (79). Another prominent difference is that cells contain a large pool of free, unconjugated SUMO2/3, but there is virtually no pool of free SUMO1, and the vast majority of SUMO1 is conjugated to proteins (80, 81). Notwithstanding the free SUMO2/3 reservoir, we noted similar SUMO1 and SUMO2/3 early responses suggesting that they can be equally dynamic. We also noted similar late responses, even the SUMO2/3 signal was remarkably stronger probably due to poly-SUMOylation. Our results show most SUMO1 conjugated at 150KDa before and after the LPS challenge. This substrate is specific as it is not observed with SUMO2/3. A common prominent band of SUMO1 Western blots described in the literature is Ran GTPase-activating Protein, RanGAP1 (90kDa) in the Nuclear Pore Complex (82). However, the SUMO1 pattern depends on the cell type, organ, and physiologic state. This SUMO1 signal at 150KDa concurs with previous studies showing similar SUMO1 patterns in HeLa cells (83). This pattern is specific to the cell types of the organ, and we didn’t see SUMO1 signal at 150KDa in other organs such as the kidney or lungs. Our results show that genetic depletion of SUMO1, but not SUMO3, mimics TAK981 treatment unleashing serum TNFα levels and HAGMA. These results show that specific SUMO1 inhibition is sufficient to unleash early complex inflammatory and metabolic responses to bacterial endotoxin. These results concur with previous studies in HEK293 cells showing SUMO1 conjugation to IκBα can prevent NF-κβ activation (84). However, SUMO regulation of NF-κβ is complex and SUMOylation can equally enhance IκBα degradation and optimize NF-κβ activity (85). SUMO1 conjugation to IκBα has not been confirmed *in vivo*, and we didn’t find a SUMO1 signal around 47KDa (IκBα molecular weight). Our results reveal three early SUMO1 signals at 100, 150, and 250KDa, of these 100KDa shows the most significant induction after the endotoxin challenge, whereas 250KDa most significant induction at 24h is associated with late stages during the resolution of inflammation versus lethal organ failure. The transfer of SUMO1-null, but not wild-type, splenocytes into splenectomized wild-type mice potentiated the inflammatory responses and mimic the results observed in global SUMO1-null mice. However, the transfer of splenocytes will not restore the complex histological and functional organization of the splenocytes, and the injection of these cells can affect peritoneal immune components. Future studies will be required for the in-depth characterization of specific cell type(s) and molecular mechanism(s) including the peritoneal immune context. Our results reveal that SUMOylation is an integral mechanism for reprograming immune and metabolic responses to infections and a potential target for controlling infectious disorders. Future specific studies will be required for the in-depth characterization of cell type(s) and molecular mechanism(s) including the specific substrates of splenic SUMO1 and their clinical implications in sepsis and cancer.

## Data availability statement

The original contributions presented in the study are included in the article/**Supplementary Material**. Further inquiries can be directed to the corresponding author.

## Ethics statement

The animal study was reviewed and approved by Duke University.

## Author contributions

LU designed and directed the study, interpreted data, trouble-shooting, and wrote the article. AY, BK and AP performed experiments, analyzed data, and prepared figures. AY, GB, WY, and AA provided reagents, training, experimental design, data interpretation, and manuscript preparation. All authors revised and approved the manuscript. All authors contributed to the article and approved the submitted version.

## Supplementary material

Supplementary Figure 1 Blood chemistry after SUMO inhibition o in endotoxemic mice. **(A)** Hematocrit, Serum levels of **(B)** potassium (K), **(C)** chloride (Cl), and **(D)** total carbon dioxide (TCO2) at 48h post-LPS (LPS, 7 mg/kg; i.p.) in control and endotoxemic mice treated with vehicle or TAK981 (7.5 mg/kg; s.c.). *P<0.05 vs Control (n=3/group, unpaired two-tailed t test).

Supplementary Figure 2 Blood chemistry of splenectomized mice after SUMO inhibition. **(A)** Hematocrit, and serum levels of **(B)** sodium (Na), **(C)** potassium (K), **(D)** chloride (Cl), **(E)** total carbon dioxide (TCO2), and **(F)** anion Gap at 48h post-LPS (LPS, 7 mg/kg; i.p.) in splenectomized (SPX) mice challenged with PBS (control) or LPS (7 mg/kg; i.p.) and treated with vehicle or TAK981.

Supplementary Figure 3 SUMOylation time-couyse during endotoxemia. Western-blots for SUMO1 **(A)** and SUMO2/3 **(B)** of spleen at the indicated post-LPS time points. Comparative densitometric analyses of **(C)** SUMO1 and **(D)** SUMO2/3 at the indicated post-LPS time-points. β-actin Western-blots (bottom panels) were used as an internal control for protein loading. were used as an internal control for protein loading. Western-blots represent experiments repeated at least twice on different days and graphs show mean ± SEM of fold of induction (FI). ***P*<*0.05 vs Control (n=3/group, two-way ANOVA with Tukey’s *post hoc* test).

Supplementary Figure 4 Immune responses in endotoxemic SUMO1-KO and SUMO3-KO mice. Western-blots analyses of **(A)** SUMO1 or **(B)** SUMO2/3 of the spleen of control and endotoxemic (LPS, 7 mg/kg; i.p.) wild-type mice treated with vehicle (WT) or TAK981 (TAK, 7.5 mg/kg; s.c.), SUMO1-KO (S1^KO^), and SUMO3-KO (S3^KO^). β-actin Western-blots are internal control for protein loading. **(C)** Serum, spleen, and liver TNFα levels at 1.5h post-LPS in endotoxemic (LPS, 7 mg/kg; i.p.) wild-type (WT), SUMO1-KO (S1^KO^), and SUMO3-KO (S3^KO^) mice. **(D)** Hematocrit, **(E)** Sodium, **(F)** chloride, and **(G)** total carbon dioxide at 48h post-LPS in wild-type (WT), SUMO1-KO (S1^KO^), and SUMO3-KO (S3^KO^). ^#^P<0.05 vs wild-type endotoxemic mice (n=6/group, Two-way ANOVA with Bonferroni’s post-hoc test).

Supplementary Figure 5 Adoptive transfer of SUMO1-null and SUMO3-null splenocytes into wild-type splenectomized mice. **(A)** Hematocrit, **(B)** Sodium, **(C)** chloride, and **(D)** total carbon dioxide at 48h post-LPS in wild-type (WT) control mice, splenectomized (SPX), and transfer of splenocytes from wild-type (SPL^WT^), SUMO1-KO (SPL^S1KO^) mice. *P<0.05 vs SPX+SPL^S1KO^ without LPS (n=6/group, unpaired two-tailed t-test).
