# Supplementary figures and images for: Splenic SUMO1 controls systemic inflammation in experimental sepsis
Source: Front Immunol. 2023 Jul 13;14:1200939. doi: 10.3389/fimmu.2023.1200939 (PMC10374847; doi:10.3389/fimmu.2023.1200939)

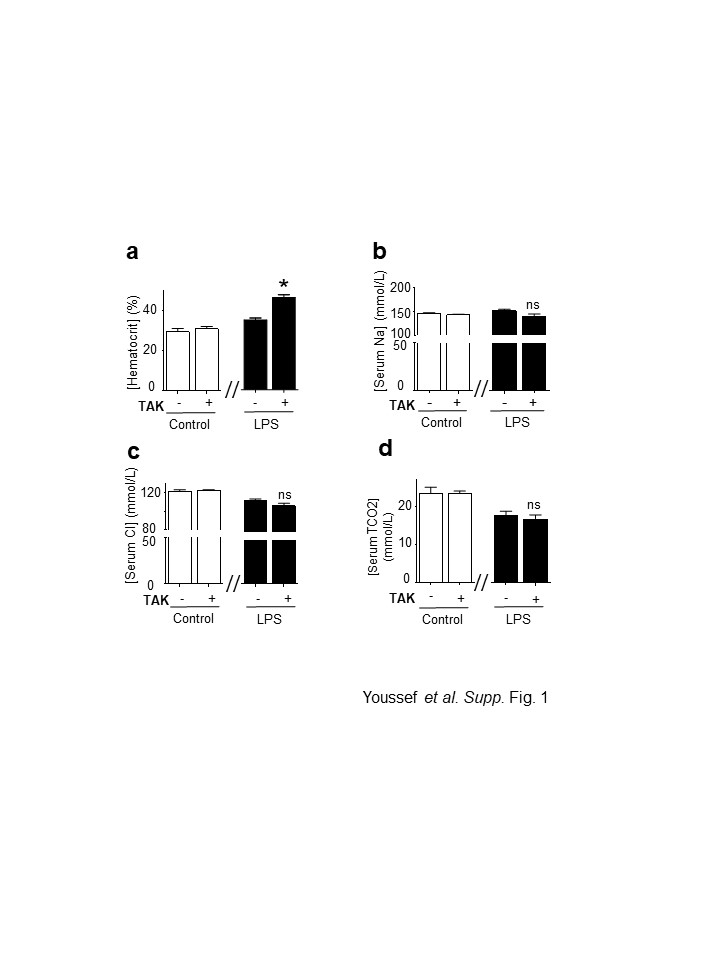

Supplement: Supplementary Figure 1 — Blood chemistry after SUMO inhibition o in endotoxemic mice. (A) Hematocrit, Serum levels of (B) potassium (K), (C) chloride (Cl), and (D) total carbon dioxide (TCO2) at 48h post-LPS (LPS, 7 mg/kg; i.p.) in control and endotoxemic mice treated with vehicle or TAK981 (7.5 mg/kg; s.c.). *P<0.05 vs Control (n=3/group, unpaired two-tailed t test). [file Image_1.jpeg]

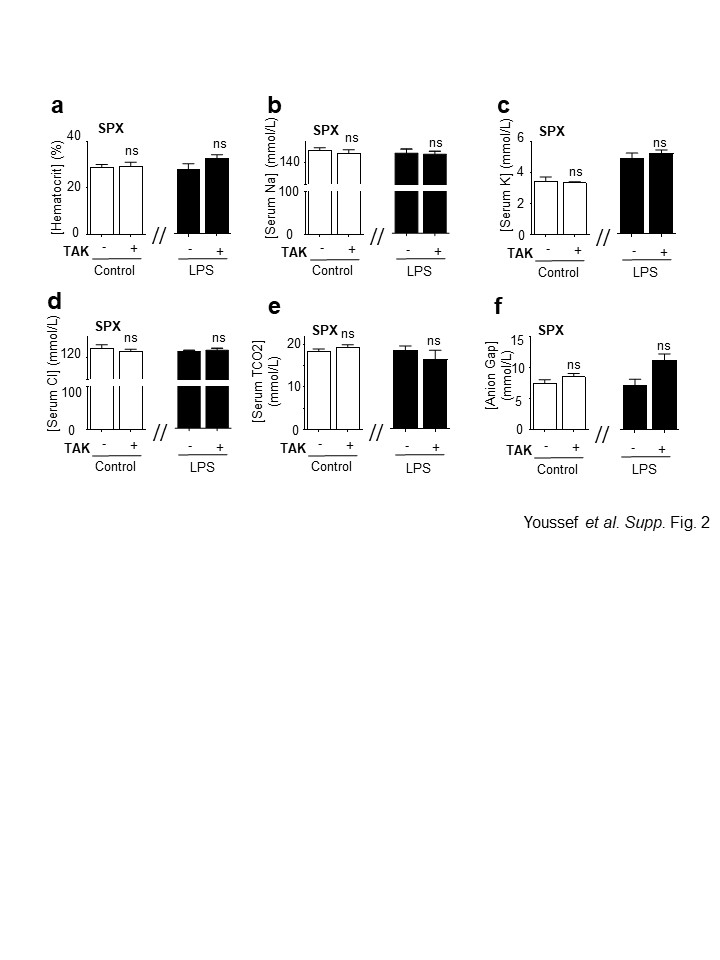

Supplement: Supplementary Figure 2 — Blood chemistry of splenectomized mice after SUMO inhibition. (A) Hematocrit, and serum levels of (B) sodium (Na), (C) potassium (K), (D) chloride (Cl), (E) total carbon dioxide (TCO2), and (F) anion Gap at 48h post-LPS (LPS, 7 mg/kg; i.p.) in splenectomized (SPX) mice challenged with PBS (control) or LPS (7 mg/kg; i.p.) and treated with vehicle or TAK981. [file Image_2.jpeg]

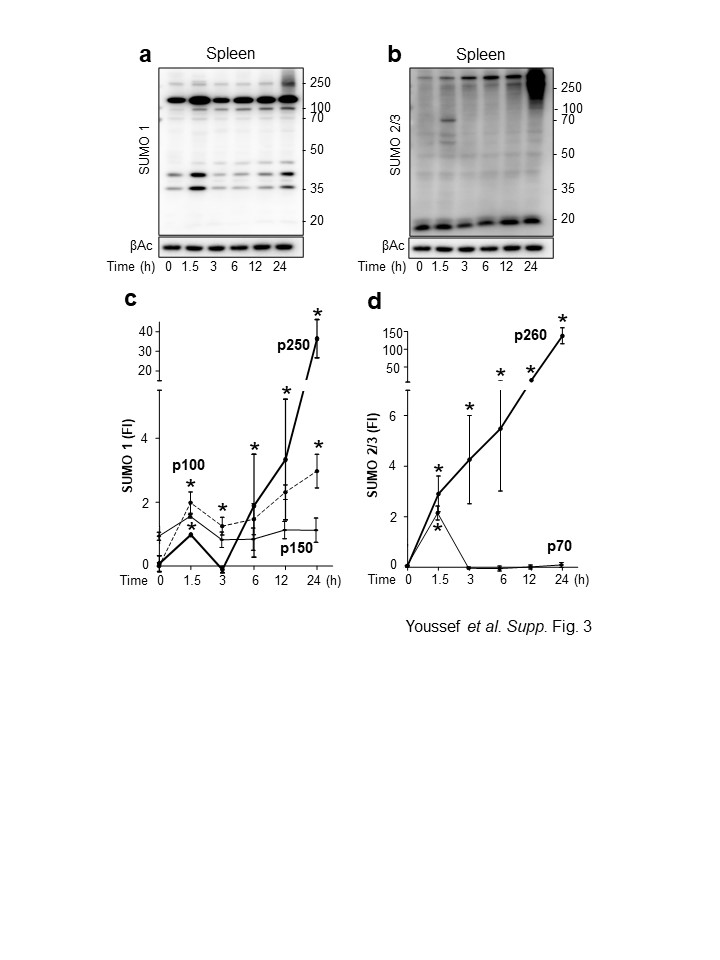

Supplement: Supplementary Figure 3 — SUMOylation time-couyse during endotoxemia. Western-blots for SUMO1 (A) and SUMO2/3 (B) of spleen at the indicated post-LPS time points. Comparative densitometric analyses of (C) SUMO1 and (D) SUMO2/3 at the indicated post-LPS time-points. β-actin Western-blots (bottom panels) were used as an internal control for protein loading. were used as an internal control for protein loading. Western-blots represent experiments repeated at least twice on different days and graphs show mean ± SEM of fold of induction (FI). *P<0.05 vs Control (n=3/group, two-way ANOVA with Tukey’s post hoc test). [file Image_3.jpeg]

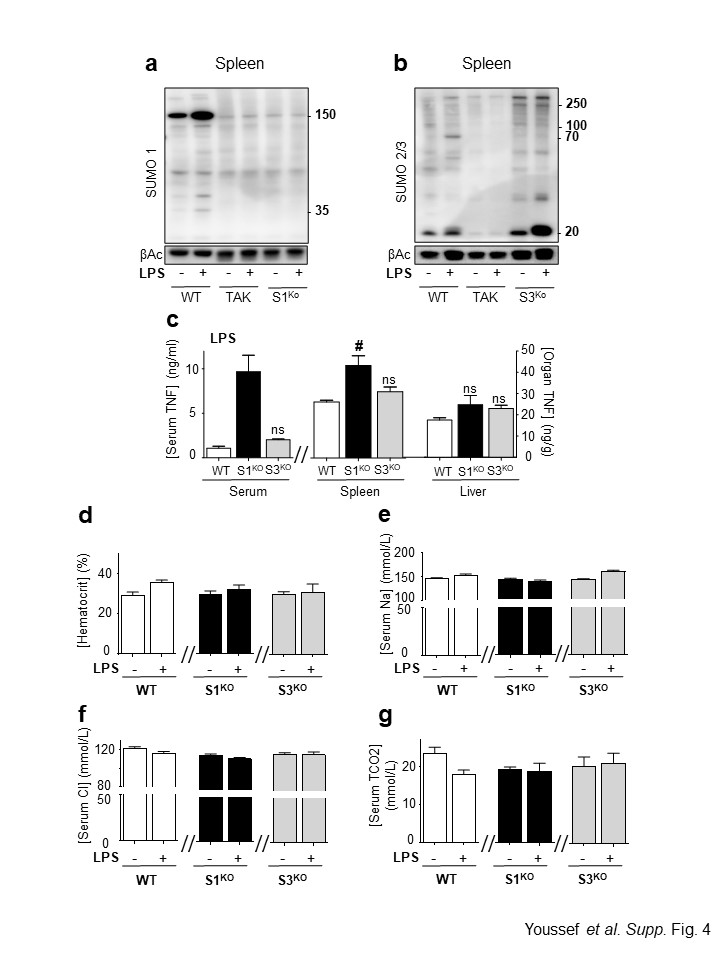

Supplement: Supplementary Figure 4 — Immune responses in endotoxemic SUMO1-KO and SUMO3-KO mice. Western-blots analyses of (A) SUMO1 or (B) SUMO2/3 of the spleen of control and endotoxemic (LPS, 7 mg/kg; i.p.) wild-type mice treated with vehicle (WT) or TAK981 (TAK, 7.5 mg/kg; s.c.), SUMO1-KO (S1KO), and SUMO3-KO (S3KO). β-actin Western-blots are internal control for protein loading. (C) Serum, spleen, and liver TNFα levels at 1.5h post-LPS in endotoxemic (LPS, 7 mg/kg; i.p.) wild-type (WT), SUMO1-KO (S1KO), and SUMO3-KO (S3KO) mice. (D) Hematocrit, (E) Sodium, (F) chloride, and (G) total carbon dioxide at 48h post-LPS in wild-type (WT), SUMO1-KO (S1KO), and SUMO3-KO (S3KO). #P<0.05 vs wild-type endotoxemic mice (n=6/group, Two-way ANOVA with Bonferroni’s post-hoc test). [file Image_4.jpeg]

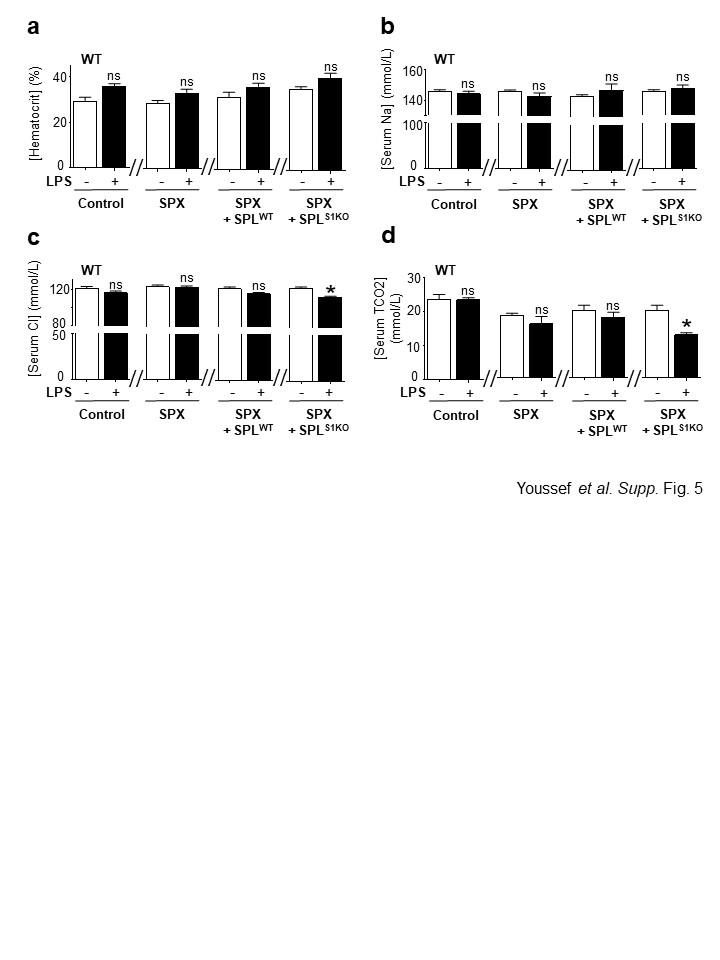

Supplement: Supplementary Figure 5 — Adoptive transfer of SUMO1-null and SUMO3-null splenocytes into wild-type splenectomized mice. (A) Hematocrit, (B) Sodium, (C) chloride, and (D) total carbon dioxide at 48h post-LPS in wild-type (WT) control mice, splenectomized (SPX), and transfer of splenocytes from wild-type (SPLWT), SUMO1-KO (SPLS1KO) mice. *P<0.05 vs SPX+SPLS1KO without LPS (n=6/group, unpaired two-tailed t-test). [file Image_5.jpeg]
